# The prediction and analysis of COVID-19 epidemic trend by combining LSTM and Markov method

**DOI:** 10.1038/s41598-021-97037-5

**Published:** 2021-08-31

**Authors:** Ruifang Ma, Xinqi Zheng, Peipei Wang, Haiyan Liu, Chunxiao Zhang

**Affiliations:** 1 School of Information Engineering, China University of Geosciences, Beijing, China; 2 School of Economic and Management, China University of Geosciences, Beijing, 100083 China; 3 Technology Innovation Center for Territory Spatial Big-Data, MNR of China, Beijing, China

**Keywords:** Diseases, Mathematics and computing

## Abstract

Corona Virus Disease 2019 (COVID-19) has spread rapidly to countries all around the world from the end of 2019, which caused a great impact on global health and has had a huge impact on many countries. Since there is still no effective treatment, it is essential to making effective predictions for relevant departments to make responses and arrangements in advance. Under the limited data, the prediction error of LSTM model will increase over time, and its prone to big bias for medium- and long-term prediction. To overcome this problem, our study proposed a LSTM-Markov model, which uses Markov model to reduce the prediction error of LSTM model. Based on confirmed case data in the US, Britain, Brazil and Russia, we calculated the training errors of LSTM and constructed the probability transfer matrix of the Markov model by the errors. And finally, the prediction results were obtained by combining the output data of LSTM model with the prediction errors of Markov Model. The results show that: compared with the prediction results of the classical LSTM model, the average prediction error of LSTM-Markov is reduced by more than 75%, and the RMSE is reduced by more than 60%, the mean ${R}^{2}$ of LSTM-Markov is over 0.96. All those indicators demonstrate that the prediction accuracy of proposed LSTM-Markov model is higher than that of the LSTM model to reach more accurate prediction of COVID-19.

## Introduction

**Figure 1:**
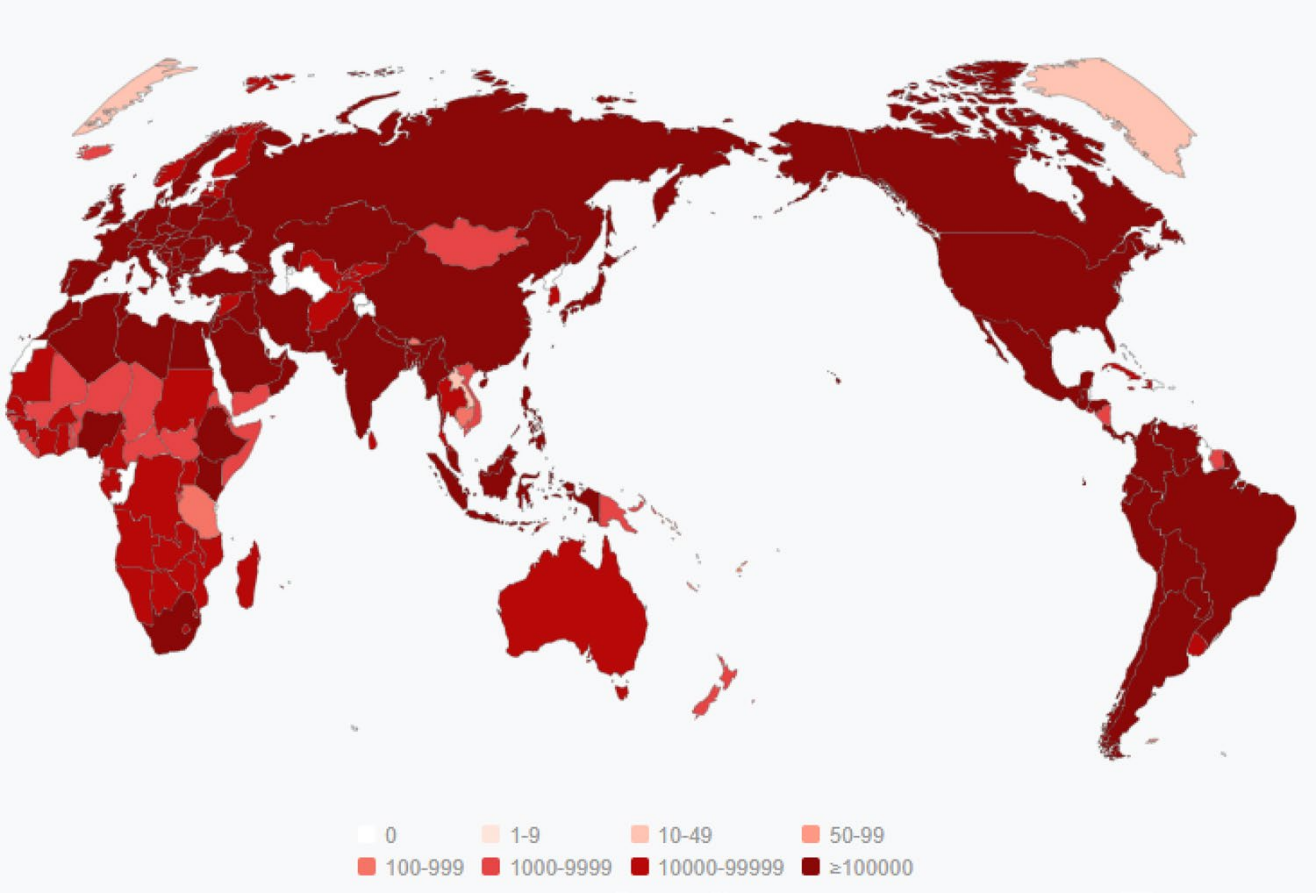
The number of people diagnosed in the world. The darker the color, the more infected people are (Map was from https://geods.geography.wisc.edu/covid19/physical-distancing/).

COVID-19 has spread to several countries around the world in a very short period and has had a huge impact on many countries. As of February 2021, more than 100 million people worldwide have been diagnosed and more than 2 million have died^1^. Unlike other infectious diseases, COVID-19 has mutated. The first wave of the epidemic broke out around March 2020. After a series of measures, the epidemic was alleviated to some extent. Since September 2020, the epidemic combined with the influenza virus broke out again^2^. In the second wave of the epidemic, the number of confirmed cases in European countries increased dramatically, which is a worrying situation. The number of diagnosed people in each country is shown in Fig. 1. Now that a vaccine has been developed, there are still many problems with the spread of vaccination^3^, we still need to minimize the spread of the disease through making policies, such as isolation, keeping a social distance and wearing a mask^4^. Therefore, predicting the future trend of the epidemic, helping relevant departments and personnel to develop policies to control the spread of the epidemic, and producing medical supplies are still extremely important.

In the field of infectious disease prediction, the main methods used could be concluded as three categories: statistics-based method, deep learning method and machine learning method. The models commonly used including the SEIR model^5^, SVM model^6^, ARIMA model^7^, LSTM model^8^, etc. For example, Kermack used epidemic model SIR to predict the development tendency of COVID-19, they believed that the transmission rate and mortality rate of the disease were fixed during the study period. However, COVID-19 did not suitable for this hypothesis^9^. Benvenuto adopted a statistical method based on moving auto-regressive model (ARIMA) to make prediction. ARIMA is a linear model, which holds that there is a linear relationship between future and past phenomena. Even though the model has a good effect in short-term prediction, it does not apply to long-term prediction of COVID-19^10^. Choi used the seasonal auto-regressive combined moving average (SARIMA) model to estimate the mortality of COVID-19^11^. Abdu Gumaei adopted a gradient enhanced regression model to estimate the mortality of COVID-19, which is a combination optimization of multiple weak regressions and can only predict a single variable^12^. All of these are statistical methods.

More and more scholars have applied deep learning methods to predict data recently. For instance, Bandyopadhyay et al. used the gate circulation network and the LSTM model to predict and estimate the number of COVID-19 diagnosed, dead and cured cases^13^. And Huang et al. used the deep learning method based on the convolutional neural network to predict the cumulative number of deaths of COVID-19^14^. Zang et al.^15^ demonstrated that CNN–LSTM, LSTM, and CNN models were more accurate than ANN and SVM models in the short-term forecasting of global horizontal irradiance (GHI). S. Bock et al.^16^ compared machine learning and deep learning models’ performance while changing the amount of input data. The results showed that the accuracy of deep learning model tend to increase as the number of training data increases. Such studies all have shown that the prediction accuracy of LSTM model will increase with the increase of training data, it can overcome the gradient vanishing and gradient explosion problems, and it has a good memory.

The purpose of this study is to develop a model that can accurately predict future epidemic trends over long periods based on historical case data, and the LSTM model still exists some problems when it comes to forecasting. For example, (1) the LSTM model uses the existing data to train model parameters, and the model parameters obtained with a large amount of existing data are accurate, otherwise, the training effect of the model may not be very well^17^. (2) The LSTM model can only predict the short-term data rather than long-term. Moreover, under limited data, the accuracy of the prediction results will also decrease with the increase of the prediction period^18^. (3) The Forget Gate in the standard LSTM model is easy to ignore and exclude relevant contents in long sequence tasks. The Forget Gate reduces the participation of previously hidden state and gives priority to calculating unit state by using the input of current state^19^. These drawbacks limit the accuracy of predictions. The improvements to the model can be divided into two categories: one is to adopt small variants of the LSTM model, that is, to improve the structure of the model itself, including Peephole connection^20^ and Gated Recurrent Unit (GRU) model^21^. The other is to combine LSTM model with other models, which typically includes the CNN-LSTM model^22^ and the SVM-LSTM model^23^, to improve the prediction accuracy of LSTM model. The above improvements to the LSTM model are all aimed at improving the accuracy of data input at the early stage of model training, so as to improve the prediction accuracy of the LSTM model. However, the disadvantage of decrease accuracy of LSTM model still remained in the long-term prediction. The Markov model is a probabilistic prediction model based on statistics, that is, the probability transfer matrix is constructed based on the data before prediction, and the probability matrix is used to predict the data^24^. The Markov model supports the detailed division of data, so Markov model can be used to correct the errors of other models, which makes up for the disadvantage that the errors of LSTM model increase with time. In view of this, the Markov model is proposed to reduce the prediction error of the LSTM model for the number of people confirmed daily, so as to improve the prediction performance of LSTM model. It is the theoretical basis for combination of the two models in this study.

The experimental results show that the combination of the LSTM and Markov model could improve the prediction accuracy of the epidemic trend effectively, and the prediction effect is also in line with reality, which has a guiding significance for the actual epidemic prediction. The contributions of this paper are summarized as below:

1. LSTM model of deep learning combined with Markov model of statistical method was designed to predict the number of confirmed cases of COVID-19.
2. The prediction errors of our proposed method (LSTM-Markov) are much smaller than that of LSTM model.
3. LSTM-Markov model can improve the accuracy and precision of medium- and long-term trend prediction of COVID-19.

## Methods

### LSTM model

**Figure 2:**
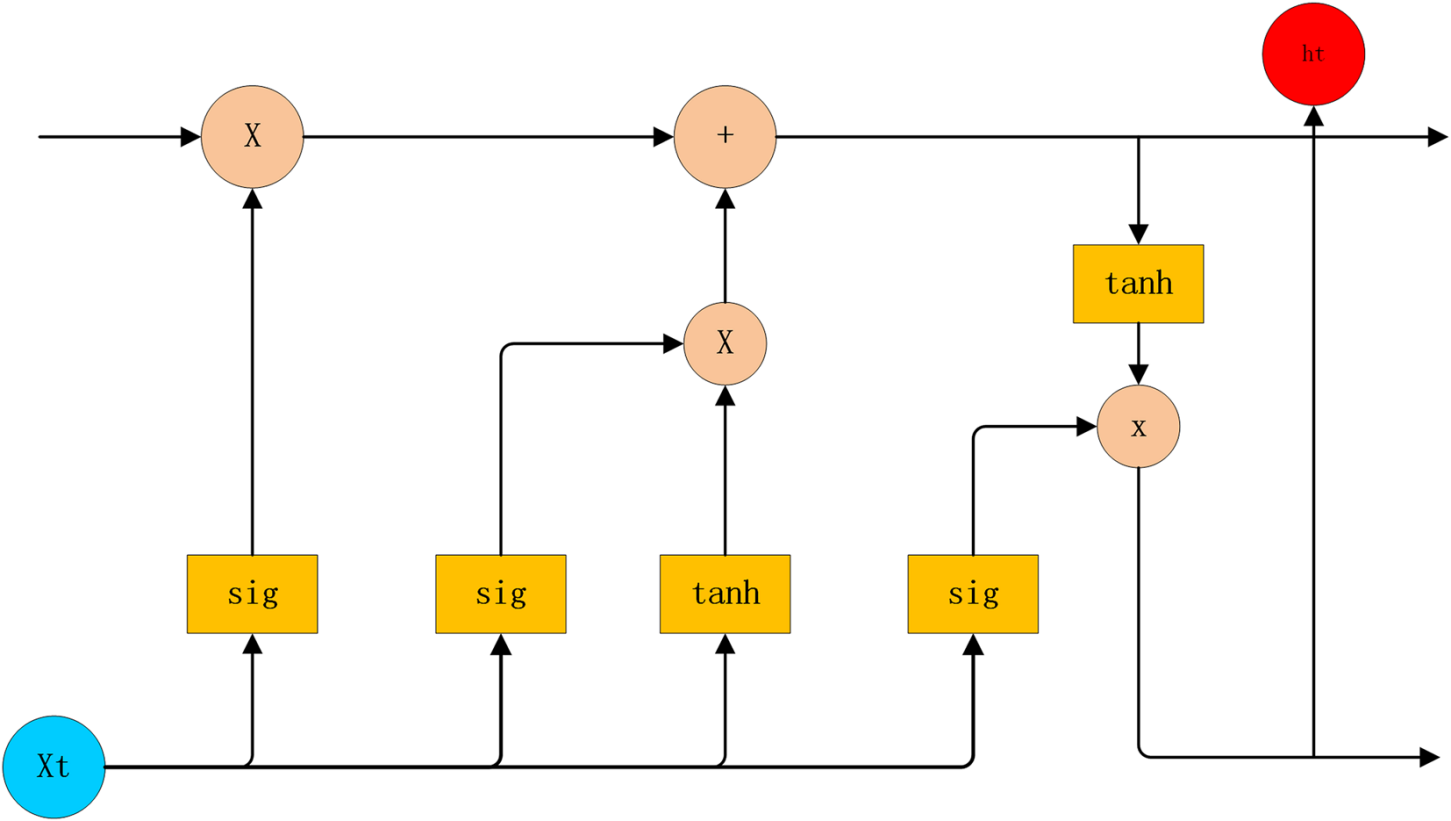
The structure of LSTM (Figure was edited by Word).

The LSTM model has been improved by the Recurrent Neural Network (RNN) and has been widely used in many fields, such as text recognition^25^, finance^26^ and industrial engineering^27^. The LSTM consists of an input layer, an output layer and hidden layers. After the input data passes through the input layer, it comes into the hidden layers. Hidden layers are the most complex and it may have multiple layers. Each hidden layer of LSTM consists of three gate units and one memory state unit. After the input information passes through three gate units and one memory unit in turn, the useful information is stored in the memory unit, and the invalid information is discarded, which can realize the prediction of the subsequent data. The function of each gate is different, and the detailed structure of the LSTM is shown in Fig. 2.

The function of each Gate in Fig. 2 can be described as follows:

1. *Forget Gate* The information first passes through the Forget Gate. The function of the Forget Gate is to determine which information from the previous layer will be discarded and which will be retained in the current state. It can be expressed as follows:
  1 $${f}_{t}=\sigma \left({W}_{f}\cdot \left[{h}_{t-1},{x}_{t} \right]+{b}_{f}\right).$$
2. *Input Gate* After entering the information, the data is updated. The Input Gate applies the $sigmoid$ function to update the data and then determines which information to store in memory cells. The specific formula is as follows:
  2 $${i}_{t}=\sigma \left({W}_{i}\cdot \left[{h}_{t-1},{x}_{t} \right]+{b}_{i}\right).$$
3. *Output Gate* The Output Gate determines the output of the model and the proportion of the output of control unit state ${C}_{t}$ to the hidden layer elements of the current LSTM model. The initial output is obtained by the $sigmoid$ activation function, then the value is reduced to – 1 to 1 by ${\text{tan}}h$ function, and then multiplies with the output of the $sigmoid$ to obtain the result, which could be expressed as follows:
  3 $${o}_{t}=\sigma \left({W}_{o}\cdot \left[{h}_{t-1},{x}_{t} \right]+{b}_{o} \right),$$
  4 $${h}_{t}={o}_{t}\cdot \text{tanh}\left({C}_{t}\right).$$
4. *Memory Cell* A line located at the top is the Memory Cell. It uses the $tanh$ function to generate new candidate values, and then combines the input information of the Input Gate with the current state information to update the memory state. It determines the information currently stored and the information transmitted to the next step, so that it can use the historical information to predict the future data. The calculation formula is as follows:
  5 $$\stackrel{\sim }{{C}_{t} }=\text{tanh}\left({W}_{c}\cdot \left[{h}_{t-1},{x}_{t} \right]+{b}_{C} \right).$$

In the above formulas, $\sigma$ presents $sigmoid$ function, ${W}_{f}, {W}_{i},{W}_{C},{W}_{o}$ represent the weight of the Forget Gate, the Input Gate, the Memory unit and the Output Gate, respectively. ${b}_{f}, {b}_{i}, {b}_{C},{b}_{o}$ represent the bias of the Forget Gate, the Input Gate, the Memory unit and the Output Gate, severally. They are all generated by random initialization function. The ${h}_{t-1}$ is the value of the hidden unit calculated at the last time, and ${x}_{t}$ is the input information at the current moment.

### Markov model

**Figure 3:**
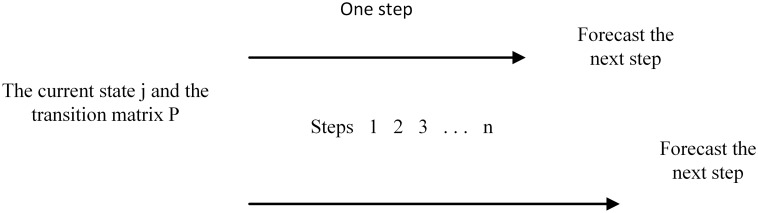
The process of Markov model (Figure was edited by Word).

The Markov is a statistical stochastic prediction model, which can be predicted only by calculating the corresponding state transition matrix according to the evolution characteristics of the event itself^28^. Markov is often used for compressing images^29^ and predicting service time of building^30^, etc. The process of Markov model is shown in Fig. 3, the principles of Markov are described as follows:

#### Definition 1

Setting up ${X}_{1},{X}_{2},\cdots {X}_{n}$ as a discrete sequence of random variables, denote as {${X}_{n}$}. All the possible values of ${X}_{n}$ are called the state space of {${X}_{n}$}, denote as $E=${${X}_{1},{X}_{2},\cdots {X}_{n}$}. If any positive integer is $n$ and any ${x}_{{i}_{1}},{x}_{{x}_{{i}_{2}}},\ldots {x}_{{x}_{{i}_{n}}}$, only if $P$(${X}_{1}={x}_{{i}_{1}},{X}_{2}={x}_{{i}_{2}}, \ldots ,{X}_{n}={x}_{{i}_{n}}$)$>0$, then,

6 $${P(X}_{n+i}={x}_{{i}_{n+1}}\left| {X}_{1}={x}_{{i}_{1}},{X}_{2}={x}_{{i}_{2}},\ldots ,{X}_{n}={x}_{{i}_{n}}\right)=P\left\{{X}_{n+1}={x}_{{i}_{n+1}}| {X}_{n}={x}_{{i}_{n}}\right\}.$$

We will call {${X}_{n}$} is a Markov chain.

#### Definition 2

Assuming that {${X}_{n}$} is the Markov chain. If any ${x}_{i},{ x}_{j}\subset E$, and if

7 $${P\{X}_{n+1}={x}_{{i}_{n+1}} \left|{X}_{n}={x}_{{i}_{n}}\right\}=P\left\{{X}_{m}={x}_{j} |{X}_{m}={x}_{i+1}\right\},$$

always is true, then we will call {${X}_{n}$} as homogeneous Markov chain.

#### Definition 3

If {${X}_{n}$} is a homogeneous Markov chain, then $P${${X}_{n+k}={x}_{j}| {X}_{n}={x}_{i}$} is called k-step transition matrix from the state ${x}_{i}$ to the state ${x}_{j}$ of {${X}_{n}$} and denoted as ${P}_{ij}\left(k\right).$ We call the matrix with ${P}_{ij}\left(k\right)$ as its elements the k-step transfer matrix of {${X}_{n}$}, recorded as ${P}_{k}$.

#### Definition 4

For any $i$, if the element ${a}_{ij}\ge 0$ of the matrix ${({a}_{ij})}_{n\times n}$, and all $\sum_{j}^{n}{a}_{ij}=1$ is true, then the matrix ${({a}_{ij})}_{n\times n}$ is a random matrix.

#### Definition 5

If matrix

8 $$A\left(n\right)=\left[\begin{array}{ccc}{a}_{11}\left(n\right)& \cdots & {a}_{1n}\left(n\right)\\ \vdots & \ddots & \vdots \\ {a}_{n1}\left(n\right)& \cdots & {a}_{nn}\left(n\right)\end{array}\right],$$

each element ${a}_{ij}\left(n\right)$ is the term of a sequence of numbers {${a}_{ij}\left(n\right)$}, then matrix $A$($n$) is called sequence matrix. And for any $i,j=\text{1,2}\cdots ,m$, if the limit of each sequence exists, we call it when the $n$ tends to infinity, $A=$(${a}_{ij}$) is the limit of $A$($n$).

According to Definition 2, if the limit matrix $P$($k$) of the k-step transition matrix of the homogeneous Markov chain exists, with the continuous evolution of the system, the transition probability between the final system states will remain unchanged, the system will show the characteristics of statistical regularity, and then it will evolve into a stable system. All systems considered in this article have a finite number of states.

### Proposed model

**Figure 4:**
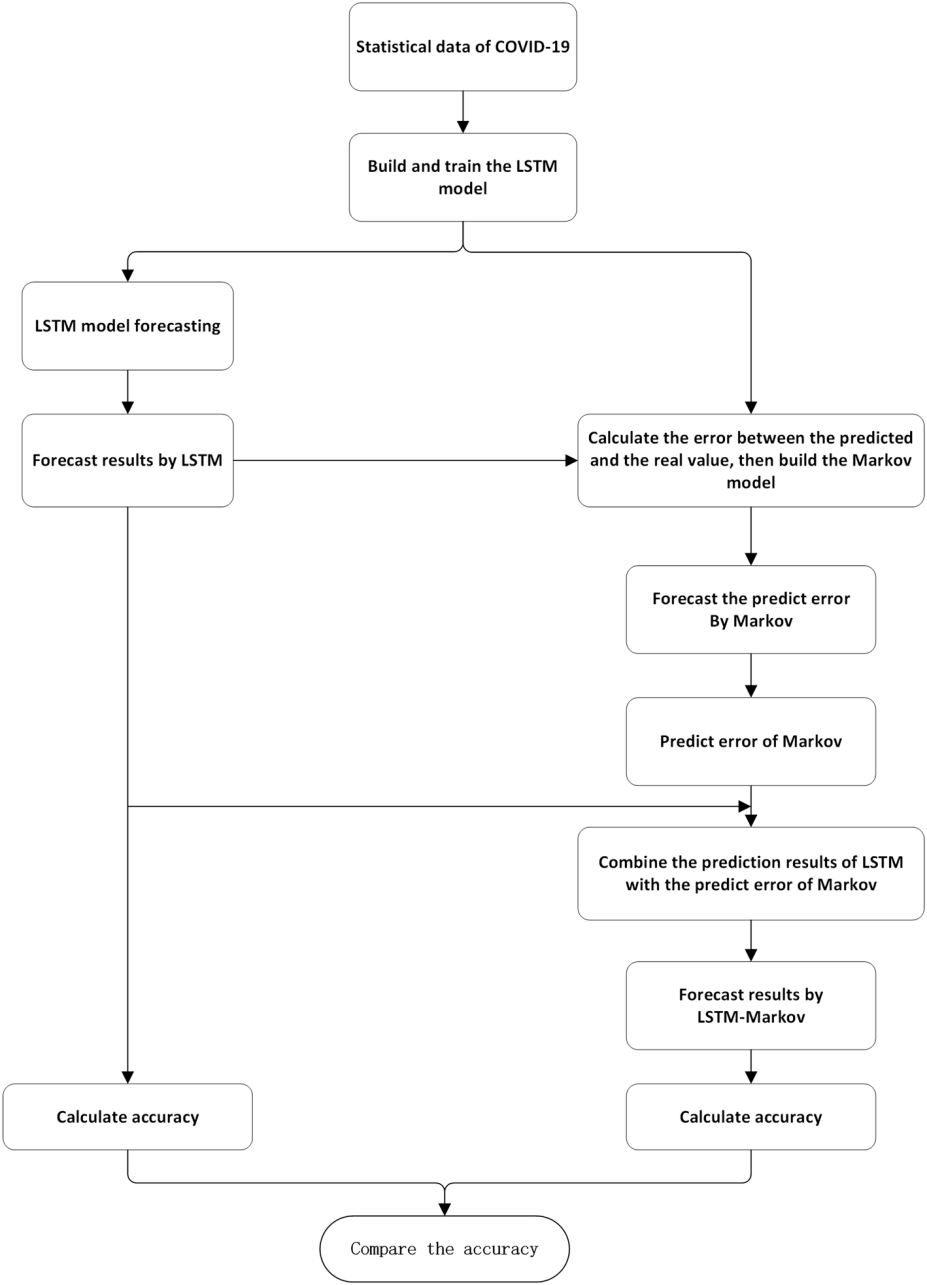
The main steps in predicting confirmed COVID-19 cases by LSTM-Markov (Figure was edited by Word).

In this study, we used Markov model to correct the prediction error of LSTM model. From reading literatures, we known that ADAM optimizer outperformed the other optimizers^31^. And to avoid over-fitting, we set the dropout to 0.02 and the hidden layer to 1 in the model^32^, and the number of nodes in the hidden layer is 4. Hence, our experiment is as follows: first, the LSTM model was trained with the confirmed cases of COVID-19 of four countries. Then, the difference between the number of confirmed cases predicted by LSTM and the actual number of confirmed cases was calculated, which was then taken as the input data of Markov model to calculate the probability transition matrix. Finally, LSTM model was used to predict the cumulative number of confirmed cases, and Markov model was used to correct the error of the prediction, so as to obtained the final forecasting results. The experimental process of our proposed method is shown in Fig. 4.

## Experiment and discussion

### Data source

The statistics used in this study were collected by John Hopkins University^33^, including four countries: the United States, Britain, Brazil and Russia, dated from March 1, 2020 to December 31, 2020. We extracted date and death data for the above countries from the repository. These four countries are the most seriously affected by the epidemic and the country with the most confirmed cases in the world. Most importantly, their curves are smooth, with no temporary surges in the middle. And the numbers of cases in these countries have been increasing, so it makes sense for us to make predictions.

### Data processing

In this study, the LSTM and LSTM-Markov models have been applied to understand the future transmission dynamics of COVID-19. The experiments are conducted on open-source libraries such as NumPy, Pandas and TensorFlow. Python, as a high-level general-purpose programming language, is used to interact with deep learning libraries as an application program interfaces (APIs). The obtained APIs is used to design the current model structure for above neural network variants.

Firstly, we divided the case data into four groups by country. Each data set for each country was considered as a time series. According to the statistical method, the data distributed outside each group data series ($\upmu -3\upsigma ,\upmu +3\upsigma$) are regarded as outliers^34^. And it’s no outliers in the four datasets. Then, the data was normalized according to the following formula:

9 $${X}_{i}=\frac{({X}_{i}-min)}{(max-min)},$$

where $min$ is the minimum value of the data and the $max$ is the maximum value of the data.

Secondly, each set of data was divided into two parts. 70% of the data were used for training the parameters of the LSTM-Markov model, and the rest of it were used for testing and prediction. The number of test days is about 100.

**Figure 5:**
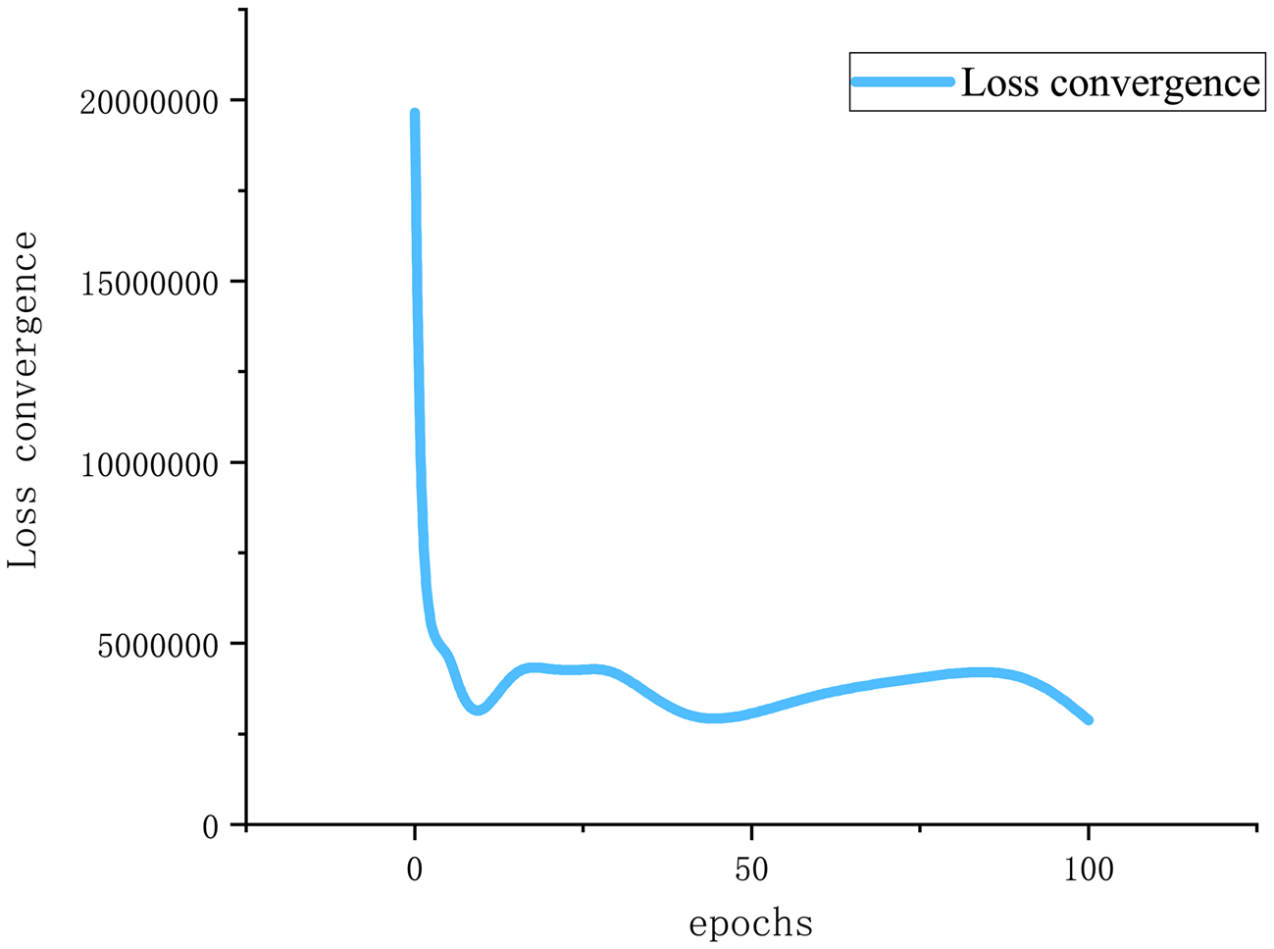
Loss convergence with different epochs.

Thirdly, setting the optimal model parameters. From reading literature, we known that the ADAM optimizer outperformed the other optimizer. So, we chosen ADAM as the model optimizer. We initially determined the range of input time step^35^, then by the trial-and-error method, we chosen the best value of window and assigned each country with corresponding best time step. The prediction effects of different parameters are shown in Tables 1 and 2. In the end, the time steps of the US, Britain, Brazil and Russia were set to 9, 7, 10 and 7 days respectively. That means: in the US, confirmed cases in the first 9 days were used to predict cases on the 10th day. In the Britain and Russia, confirmed cases in the first 7 days were used to predict cases on the 8th day. In the Brazil, the number of days to input is 10. For the epochs, as shown in Fig. 5, when the epoch is 50, loss convergence is the minimal. So, 50 is also more appropriate. With the optimal parameters, the resulting model is also optimal in weights and biases. Tables 1, 2 and 3 show the setting of model parameters in the four countries:

**Table 1:** The model’s RMSE of different time span.

US			Britain			Brazil			Russia		
Timespan	LSTM RMSE	LSTM-Markov RMSE	Timespan	LSTM RMSE	LSTM-Markov RMSE	Timespan	LSTM RMSE	LSTM-Markov RMSE	Timespan	LSTM RMSE	LSTM-Markov RMSE
3	3,271,974	2,204,629	3	426,583	248,493	3	698,082	506,549	3	586,483	139,460
4	4,298,475	1,818,030	4	239,493	485,919	4	807,812	422,905	4	381,164	139,842
5	4,298,801	2,755,216	5	421,336	692,762	5	1,028,703	590,634	5	169,464	246,306
6	4,533,149	2,636,602	6	219,766	306,029	6	884,107	333,420	6	532,194	210,934
7	4,659,424	2,957,439	7	331,109	236,511	7	1,105,731	431,629	7	440,670	89,942
8	4,465,570	3,037,668	8	344,832	579,751	8	726,134	245,414	8	464,402	338,961
9	3,006,406	1,249,420	9	440,334	244,718	9	760,957	379,625	9	476,284	252,601
10	3,418,221	2,920,985	10	358,119	754,001	10	432,045	199,163	10	303,172	238,583
11	5,026,319	2,632,837	11	715,564	257,792	11	742,365	555,268	11	369,349	327,625
12	4,912,377	4,251,474	12	410,471	380,434	12	1,072,279	723,964	12	245,291	312,600
13	4,501,122	2,717,536	13	985,517	258,268	13	906,820	380,523	13	520,249	140,814
14	4,365,950	2,889,534	14	468,084	305,483	14	1,033,211	414,927	14	76,835	143,322
15	3,773,912	3,775,956	15	526,129	1,314,939	15	953,686	322,795	15	285,784	198,222

**Table 2:** The model’s R2 of different time step.

US			Britain			Brazil			Russia		
Time span	LSTM R2	LSTM-Markov R2	Time span	LSTM R2	LSTM-Markov R2	Time span	LSTM R2	LSTM-Markov R2	Time span	LSTM R2	LSTM-Markov R2
3	0.67	0.85	3	0.81	0.87	3	0.72	0.85	3	0.33	0.96
4	0.42	0.9	4	0.94	0.75	4	0.62	0.9	4	0.71	0.96
5	0.41	0.76	5	0.81	0.49	5	0.37	0.79	5	0.94	0.88
6	0.33	0.77	6	0.95	0.9	6	0.53	0.93	6	0.41	0.91
7	0.28	0.71	7	0.88	0.94	7	0.26	0.89	7	0.59	0.98
8	0.33	0.69	8	0.87	0.63	8	0.67	0.96	8	0.53	0.75
9	0.69	0.96	9	0.78	0.93	9	0.64	0.91	9	0.5	0.86
10	0.59	0.7	10	0.86	0.36	10	0.88	0.97	10	0.79	0.87
11	0.09	0.75	11	0.42	0.92	11	0.64	0.8	11	0.68	0.75
12	0.11	0.34	12	0.81	0.83	12	0.24	0.66	12	0.86	0.77
13	0.24	0.72	13	− 0.12	0.92	13	0.45	0.9	13	0.34	0.95
14	0.27	0.68	14	0.74	0.89	14	0.27	0.88	14	0.99	0.95
15	0.44	0.44	15	0.67	− 1.05	15	0.37	0.93	15	0.79	0.9

**Table 3:** Final determined model parameters.

Country	Layers number	Units number	Time step	Dropout	Epochs
US	1	4	9	0.02	50
Britain	1	4	7	0.02	50
Brazil	1	4	10	0.02	50
Russia	1	4	7	0.02	50

Finally, the trained LSTM model and the LSTM-Markov model were used to predict the number of daily confirmed cases in each country before February 20, 2021, respectively.

### Assessment indicator

There are errors between predicted data and actual data. In this paper, RMSE (root-mean-square error) was used to evaluate the degree of dispersion of error. In order to evaluate the fitting degree of models, we chosen ${R}^{2}$ (R-squared) index, and we used the error rate to evaluate the accuracy of the prediction, which are defined as follows:

10 $$RMSE=\sqrt{\frac{1}{n}\sum_{i=1}^{n}\left(y-\widehat{y}\right),}$$

11 $${R}^{2}=1-\frac{\sum {\left(y-\widehat{y}\right)}^{2}}{\sum {\left(y-\overline{y }\right)}^{2}}, $$

12 $$Error \,rate=\frac{\left|y-\widehat{y}\right|}{y}, $$

where $y$ is the true value, $\widehat{y}$ is the predicted value, $n$ is the number of values.

Root mean square error (RMSE) of the LSTM model and the improved model proposed in this paper were compared to determine whether the prediction accuracy of the model was improved^36,37^. The smaller the value of RMES, the better the performance. The ${R}^{2}$ was used to evaluate the fitting degree of the two models^38^, the closer to 1, the better the model works. The $error rate$ was used to estimate the accuracy of prediction, the closer to 0, the more accurate.

### Experimental results

**Figure 6:**
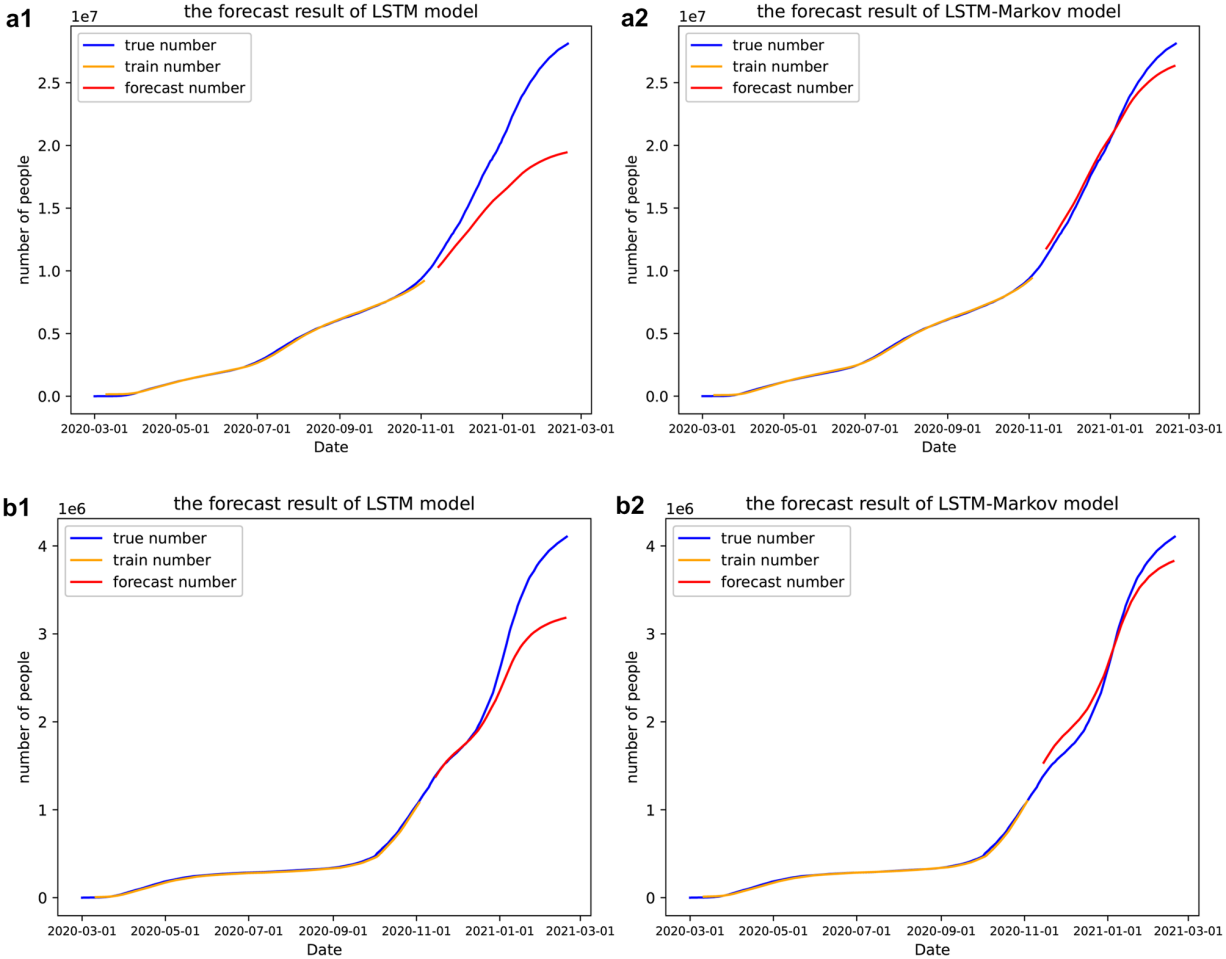

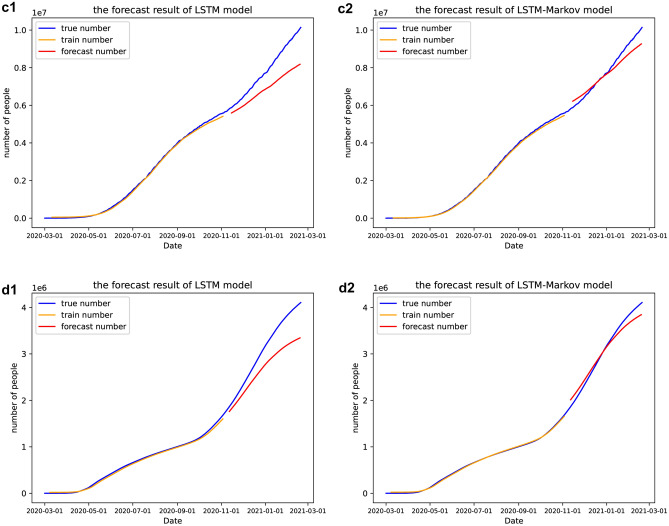
Prediction curves of cumulative number of COVID-19. (**a**) In the United States, (**b**) in Britain, (**c**) in Brazil, (**d**) in Russia. The blue line represents the number of reported confirmed cases, the orange line is the curve of the cumulative confirmed cases we trained, and the red line is the curve of forecasting. 1 represents the results of LSTM model, while 2 represents the results of LSTM-Markov model. (Figure was from the python 3.6.).

In this paper, LSTM model and the proposed LSTM-Markov model were applied to predict the number of daily total infected cases of COVID-19 in the four countries mentioned above respectively and the results are shown in Fig. 6.

As can be seen from Fig. 6, the curves keep rising as time goes on, especially after October 2020, the curves rise steeply. This implies that the situation became more severe in October. We predicted that by January 2021, the Britain will stabilize to 3.5 million. Then its epidemic will be brought under control. In the US and Russia, the number of daily confirmed cases would still see further increase, but the curves were starting to flatten and the growth would slow down around February. While Brazilian cases would continue to see rapid increases, with no signs of slowing down. We predict that more than 8 million people infected by February 2021.

**Figure 7:**
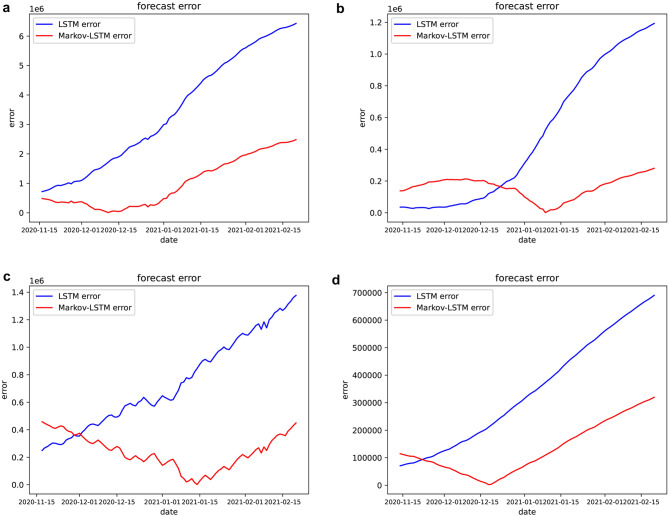
Comparison of forecast errors by countries. (**a**) In the United States, (**b**) in Britain, (**c**) in Brazil, (**d**) in Russia. The blue line represents the results of the LSTM model, the red line represents the results of the LSTM-Markov model (Figure was edited by the python).

In addition, the prediction errors of LSTM model and LSTM-Markov model were calculated and compared, as is shown in Fig. 7.

According to Fig. 7, the prediction errors of LSTM model increase very fast, and the errors increase the fastest at about 30 days. In the US, the prediction errors of the LSTM-Markov model are always smaller than the LSTM model. In other countries, the errors of LSTM-Markov model are slightly larger than LSTM in the initial stage, but far less than that of LSTM in the middle and late stage. By February 2021, the errors of the LSTM-Markov model are less than that of the LSTM model 4 million in the US, 1 million in the Britain and Brazil and 40,000 in the Russia, respectively. The result indicates that the proposed LSTM-Markov model greatly reduces the prediction error of the LSTM model.

**Figure 8:**
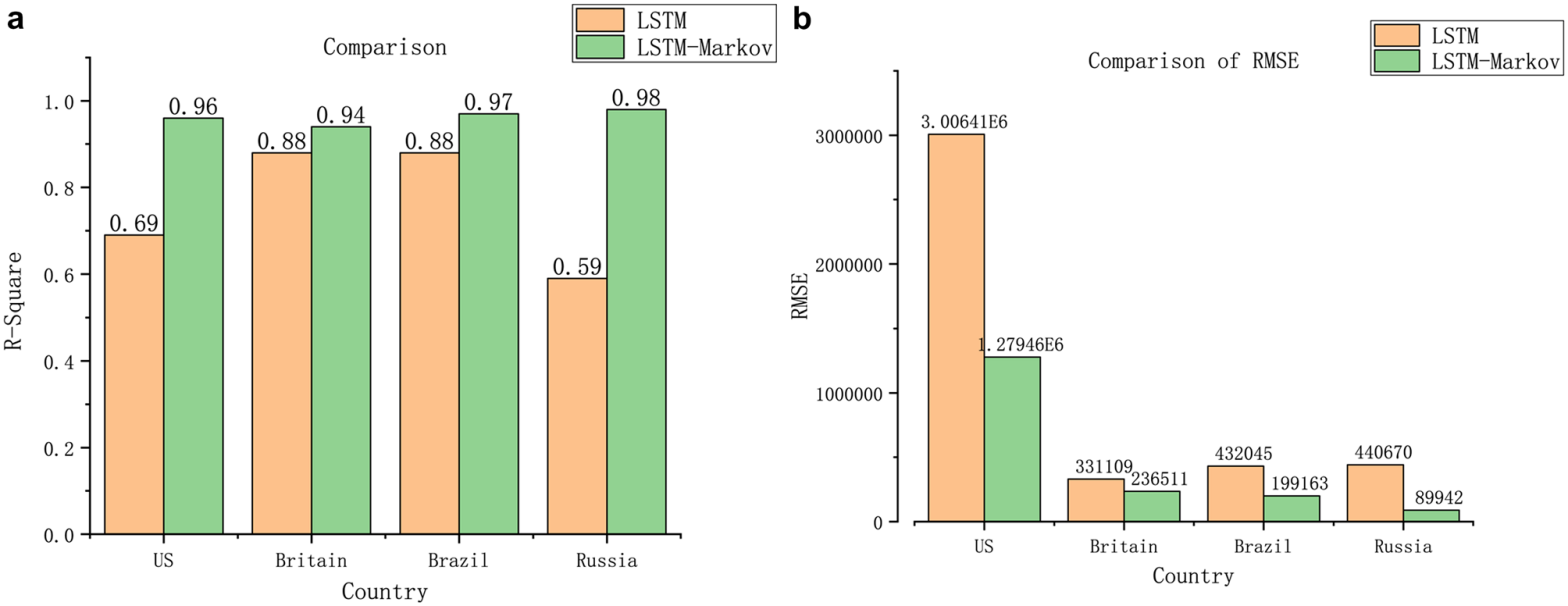
Comparison of ${R}^{2}$ and RMSE between LSTM and LSTM-Markov model, a represents ${R}^{2}$, and b represents RMSE (Figure was edited by Origin).

We calculated RMSE and ${R}^{2}$ of the LSTM model and the LSTM-Markov model respectively, which are shown in Fig. 8.

**Figure 9:**
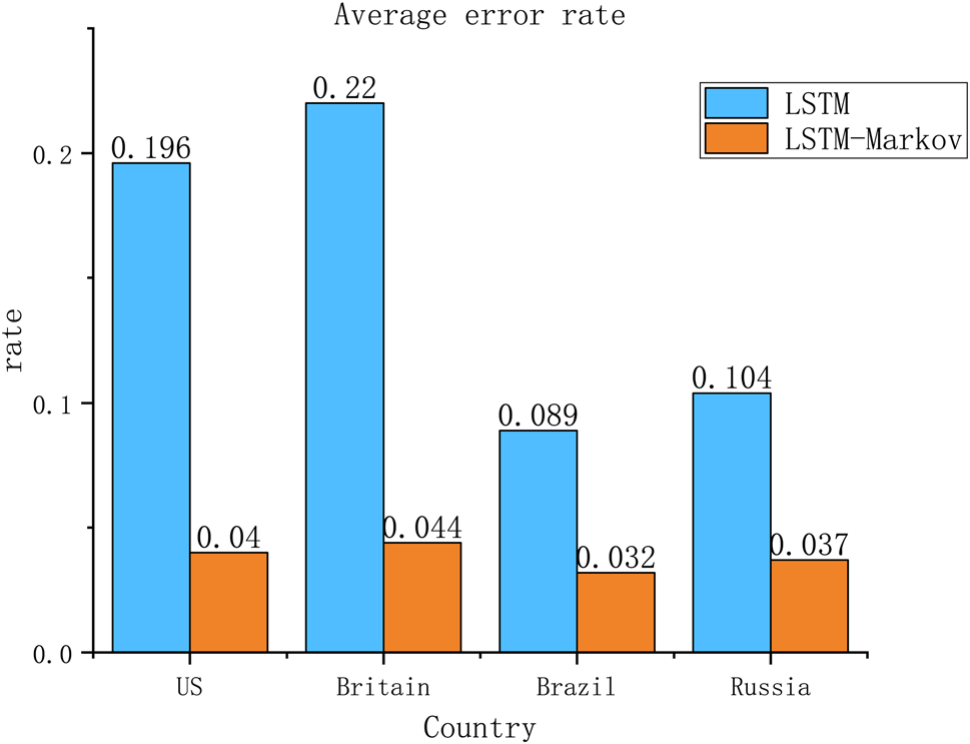
Comparison of average error rate between the LSTM and LSTM-Markov model (Figure was edited by Origin).

To verify the effectiveness of our proposed method, the cumulative number of infected cases predicted by the two models for December 5, 2020, January 5, 2021 and February 5, 2020 were compared with the real values, respectively. As shown in Table 4 and Fig. 9.

**Table 4:** Comparison of cumulative confirmed cases reported and predicted on a daily basis.

Country	Date	Reported value	LSTM predicted value	LSTM error rate	LSTM-Markov predicted value	LSTM-Markov error rate
US	2020.12.5	14,733,807	13,329,365	0.095	14,564,888	0.011
	2021.1.5	21,182,522	15,354,448	0.275	20,506,525	0.032
	2021.2.5	26,879,739	21,051,665	0.217	24,782,471	0.078
Britain	2020.12.5	1,705,971	1,650,785	0.032	1,492,824	0.125
	2021.1.5	2,774,479	2,253,392	0.188	2,773,468	0.001
	2021.2.5	3,911,573	2,187,127	0.441	3,883,746	0.007
Brazil	2020.12.5	6,577,177	6,139,593	0.067	6,271,946	0.046
	2021.1.5	7,810,400	7,197,898	0.078	7,625,120	0.024
	2021.2.5	9,447,165	8,288,897	0.123	9,191,593	0.027
Russia	2020.12.5	2,629,699	2,487,484	0.054	2,576,114	0.02
	2021.1.5	3,250,713	2,907,795	0.105	3,162,229	0.027
	2021.2.5	3,891,274	3,302,068	0.151	3,638,792	0.065

As can be seen from a in Fig. 8, in the US, Britain, Brazil and Russia, the ${R}^{2}$ of LSTM-Markov are 0.96, 0.94, 0.97 and 0.98, with the average value greater than 0.96 and close to 1, both are larger than LSTM model. So, we can know that the proposed model has better fitting effect than the LSTM model. From b in Fig. 8, the RMES of LSTM-Markov model is nearly 40% of LSTM, which proved that the forecasting precision is greatly improved by of LSTM-Markov model. According to Fig. 9, compared with the number of reported cases, the average LSTM-Markov error rates for the US, Britain, Brazil and Russia were 0.040, 0.044, 0.032 and 0.037, respectively. Its average prediction error rate was 0.038 and the average error rate of LSTM is 0.152. As a result, the error was reduced by more than 75%, far less than the LSTM model, and the accuracy was improved by 60%. Both the short-term and long-term prediction error rates of LSTM-Markov model are lower than the LSTM model.

## Discussion

As can be seen from Fig. 6, the prediction curve of our proposed model has the same trend as the actual curves, and is closer to the real curve than the prediction curve of the LSTM model. We predict that the number of cases will continue to increase in these countries, and then in January 2021, the curve of cumulative confirmed cases will be gradually slow in the Britain, the number of cases will stabilize at about 3.5 million, so, the epidemic will be brought under control. And the number of cases will continue to increase in US, Russia and Brazil, but Brazil’s growth will not slow. It can be seen from Figs. 7, 9 and Table 2 that the prediction error curve of the LSTM-Markov model is much lower than the LSTM model. The average error rate of the LSTM model is 0.152, while the average error rate of LSTM-Markov model is 0.038. Both the short-term and long-term prediction error rates of the LSTM-Markov model are smaller than those of the LSTM model. The Fig. 8 show that the prediction accuracy of the LSTM-Markov model is much higher than that of the LSTM model through the ${R}^{2}$ and RMSE value.

After the new president of the US took office, he paid special attention to epidemic prevention. He signed an executive order requiring the nation to wear masks and issued a quarantine order. He announced that the national strategy will be driven by scientists and public health experts who will communicate directly to you^39^. The United States began to gradually lift the blockade recently, and has distributed nearly 4 million vaccines to the country by February 2021. The vaccine acceptance rate in the US is 56.9%^40^. We can also read from Fig. 6 that the number of people diagnosed in the United States increased rapidly in January and gradually slow at the end of January, indicating the effectiveness of the U.S. policy. The British government has also taken a lot of treasures to control the epidemic. The National Health Service (NHS) has handed out £4.2 million in December 2020 to vaccinate the groups most in need and reduce vaccine inequality^41^. And the UK has committed to rolling out vaccines as a top priority for caring for residents and staff. Since the new year, the delivery system in England comprises the original hospital hubs and primary care services, now supplemented by mass vaccination centers and community pharmacy services. By the end of January, more than 300,000 vaccinations were being given each day^42^. In conclusion, what we see from our experimental results is that in February, the number of diagnoses gradually slowing down in both countries and the epidemic was brought under control, which is consistent with what we predicted.

The Russian government did not pay enough attention to COVID-19 in the early days, leading to a rapid outbreak. Later, due to the abolition of unprofitable hospitals, polyclinics and infectious disease beds, the shortage of doctors and the heavy workload of medical institutions, therefore, the number of confirmed cases in Russia will continue to increase for some time to come^43^. And in Brazil, the governmental response to COVID-19 has been marked by the lack of leadership at the federal level, distrust of science, denial of the importance of the virus and progressive cuts to health and research funding. There are racial and gender differences in the fight against novel corona-virus^44^. Brazil, of course, has by far the worst outbreak, and the number of confirmed cases is still rising dramatically, at the same time our experimental results also indicate this point.

## Summary

COVID-19 has been announced as a global pandemic, and has drawn great attention of countries all over the world. This study proposes a LSTM model combined with Markov model (LSTM-Markov) in view of the traditional LSTM models predict problems deviation of the data. First, the model was trained by confirmed case data from four countries: the US, Britain, Brazil and Russia. Then, predicting the number of confirmed cases before February 20, 2021 in each country by using the Markov model correcting LSTM model. Finally, using ${R}^{2}$, RMSE value and $error\, rate$ to evaluate the effectiveness of our proposed model.

We predicted that the number of cases will stabilize and the epidemic will be brought under control in the Britain by February 2021, while the number of cases will continue to rise in US, Brazil and Russia. The results show that the prediction curve of the proposed LSTM-Markov model is closer to the real epidemic curve, the mean RMSE is only 40% of the LSTM model, the ${\text{R}}^{2}$ are all close to 1, the average error is reduced by more than 75%. Thus, the forecasting accuracy of LSTM-Markov is far higher than LSTM model. By comparing the $error \,rate$ of LSTM-Markov model with LSTM model, the results show that the former has better prediction effect. And compared with other research results^45–47^, our improvement of LSTM model is better. In conclusion, LSTM-Markov model can predict the confirmed cases effectively, the predicted results can also provide help and reference for the government decision-making in formulating relevant measures, and have practical significance in life.

### Threads

However, this method still has some shortcomings. We didn’t experiment with more countries to see if the model works for all countries. Later, if possible, we will apply the model to other countries to improve the model. And the influencing factors only include the number of confirmed cases, without considering various influencing factors such as gender, age, occupation or location. In the future, we will continue to improve the model and add a variety of influencing factors in the later stage to further improve the accuracy of prediction.

## Data Availability

The datasets generated during and/or analyzed during the current study are available in the GitHub repository [https://github.com/CSSEGISandData/COVID-19].
